# Emergence of number sense through the integration of multimodal information: developmental learning insights from neural network models

**DOI:** 10.3389/fnins.2024.1330512

**Published:** 2024-01-17

**Authors:** Kamma Noda, Takafumi Soda, Yuichi Yamashita

**Affiliations:** Department of Information Medicine, National Institute of Neuroscience, National Center of Neurology and Psychiatry, Kodaira, Japan

**Keywords:** deep learning, representation learning, multimodal learning, sensory integration, numerosity, mathematical ability

## Abstract

**Introduction:**

Associating multimodal information is essential for human cognitive abilities including mathematical skills. Multimodal learning has also attracted attention in the field of machine learning, and it has been suggested that the acquisition of better latent representation plays an important role in enhancing task performance. This study aimed to explore the impact of multimodal learning on representation, and to understand the relationship between multimodal representation and the development of mathematical skills.

**Methods:**

We employed a multimodal deep neural network as the computational model for multimodal associations in the brain. We compared the representations of numerical information, that is, handwritten digits and images containing a variable number of geometric figures learned through single- and multimodal methods. Next, we evaluated whether these representations were beneficial for downstream arithmetic tasks.

**Results:**

Multimodal training produced better latent representation in terms of clustering quality, which is consistent with previous findings on multimodal learning in deep neural networks. Moreover, the representations learned using multimodal information exhibited superior performance in arithmetic tasks.

**Discussion:**

Our novel findings experimentally demonstrate that changes in acquired latent representations through multimodal association learning are directly related to cognitive functions, including mathematical skills. This supports the possibility that multimodal learning using deep neural network models may offer novel insights into higher cognitive functions.

## 1 Introduction

The integration of multimodal information is essential for human cognitive abilities. We perceive our environment by the integration of various types of input from multiple sources such as vision, sound, and touch to achieve appropriate cognitive behavior. For example, higher-order brain functions such as language, semantic memory, and calculation inevitably involve multimodal association (MMA; Büchel et al., 1998; Kraut et al., 2002). Additionally, while MMA provides considerable advantages for higher cognitive function, its failure results in a wide range of neuropsychological symptoms associated with neurodevelopmental disorders and neurodegeneration. For example, the core pathologies of semantic dementia (Nishio and Mori, 2009), calculation disorder (Rousselle and Noël, 2007), and prosopagnosia (Gainotti and Marra, 2011) are considered to be due to alterations in MMA. However, despite intensive investigations (Andersen, 1997; Ardesch et al., 2019), researchers have not yet established a definitive MMA theory. Understanding the fundamental process of MMA could reveal the underlying mechanisms of the human brain and intelligence, and also contribute to understanding the pathologies and prevention of neurodevelopmental disorders and neurodegeneration.

Mathematical ability is a representative example of a cognitive process related to MMA. Number sense (numerosity), which involves the ability to judge the magnitude of numbers, may form the basis of mathematical skills (Nieder and Miller, 2003; Dehaene, 2011). MMA, such as linking number sense with numerical symbols, is considered a crucial element in this process (Verguts and Fias, 2004; Diester and Nieder, 2007; Gevers et al., 2016). Indeed, Parham (1998) reported that the sensory integration ability, assessed through multiple tests measuring coordination across various sensory modalities, is associated with arithmetic achievement in children. Moreover, individuals with reduced ability in mathematics exhibited lower performance in tasks involving symbolic numbers rather than non-symbolic numbers (Rousselle and Noël, 2007). Although these studies imply the importance of MMA in the development of mathematical ability, the neural systems supporting mathematical skills and the contributions of MMA are not well understood. One major barrier to this understanding is the technical challenges associated with deciphering the intricate neural underpinnings of MMA. The complexity of brain network interactions and multilayered nature of cognitive processing make it difficult to isolate and study the precise neural systems that are involved.

Given these challenges, computational modeling using artificial neural networks has emerged as a promising approach. This method contributes to the understanding of cognitive abilities across multiple scales, including neurons, circuits, and cognition. For example, several studies have reported similarities between deep neural networks and the human brain (Serre, 2019; Sinz et al., 2019; Yang and Wang, 2020). Additionally, the concept of multimodal learning has been applied in the fields of machine learning and deep neural networks (Baltrušaitis et al., 2019; Suzuki and Matsuo, 2022). Learning by using multiple modalities enhances the performance of neural network models (Shi et al., 2019).

Another key aspect of studying deep neural networks is the acquisition of better latent representations (Bengio et al., 2013; Lu et al., 2017; Tschannen et al., 2018). Latent representations based on multimodal information are believed to play an important role in achieving superior performance (Guo et al., 2019). We hypothesize that changes in latent representations acquired through MMA are related to differences in cognitive abilities, including mathematical skills. Previous studies have reported that neural networks acquire a latent space that reflects number sense (Stoianov and Zorzi, 2012; Zorzi and Testolin, 2017; Di Nuovo and Jay, 2019; Testolin et al., 2020; Kim et al., 2021). For example, the neural response in an artificial neural network, when presented with dot stimuli representing varying numbers, replicates the tuning curves of neurons in monkeys (Nasr et al., 2019). Furthermore, neural network models using multimodal information are reportedly effective in the acquisition of mathematical abilities (Verguts and Fias, 2004; Di Nuovo and McClelland, 2019; Sabathiel et al., 2020). However, these studies did not explore how representations related to numerosity are acquired by integrating multimodal information, and did not investigate the potential impact of these representations on subsequent mathematical tasks.

We aimed to use a multimodal deep neural network model as a computational model for MMA in the brain. We sought to determine the mechanisms underlying the effect of multimodal learning on the representation of information and its influence on cognitive task performance. In the experiment, we compared the representations of numerical information, i.e., handwritten digits and images containing a variable number of geometric figures learned in single and multimodal ways, which assumably corresponded to the human cognitive process of learning number sense. We tested this hypothesis through a subsequent experiment that investigated the effect of changes in representation on the performance of a downstream arithmetic task.

## 2 Materials and methods

### 2.1 Task

In this experiment, we targeted two tasks: a reconstruction task and a cross-generation task, in order to observe changes in latent representations when performing both tasks simultaneously or only the reconstruction task.

**Figure 1:**
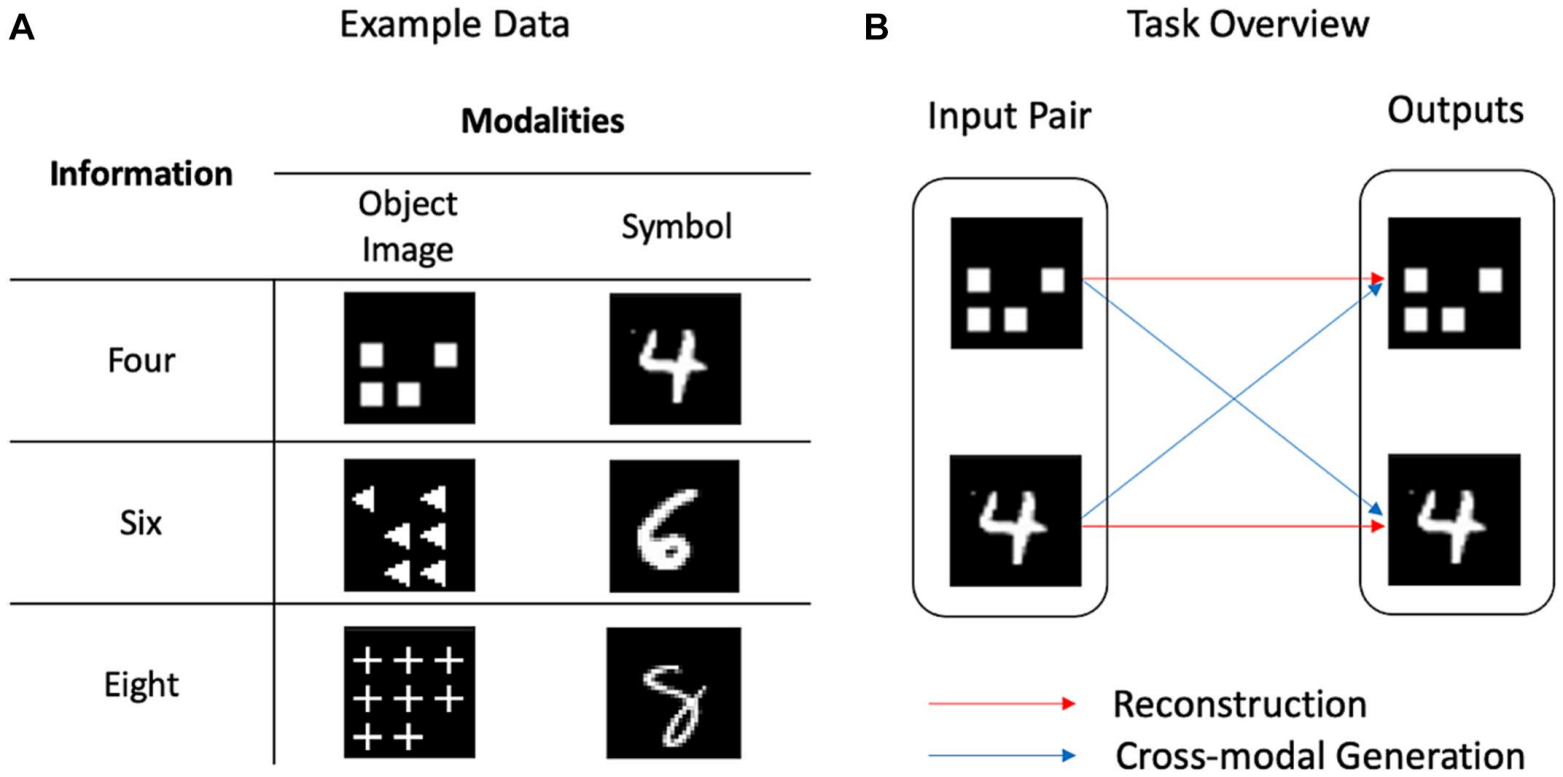
Example data and task overview. **(A)** Identical information could be presented in different ways. As an object image, the number four is presented as an image of four squares, whereas it is presented as the handwritten digit “4” using the symbol. **(B)** Reconstruction refers to the generation within one modality, whereas cross-modal generation refers to the generation across several modalities.

Figure 1 presents an overview of these tasks. Reconstruction refers to generating data within one modality (e.g., generating a symbol similar to the input symbol), whereas cross-modal generation refers to generating data with identical information as the input, but with different modalities (e.g., generating an image of three objects for a symbol of three). Performing the reconstruction and cross-modal generation tasks together presumably models MMA in the human cognitive process.

**Figure 2:**
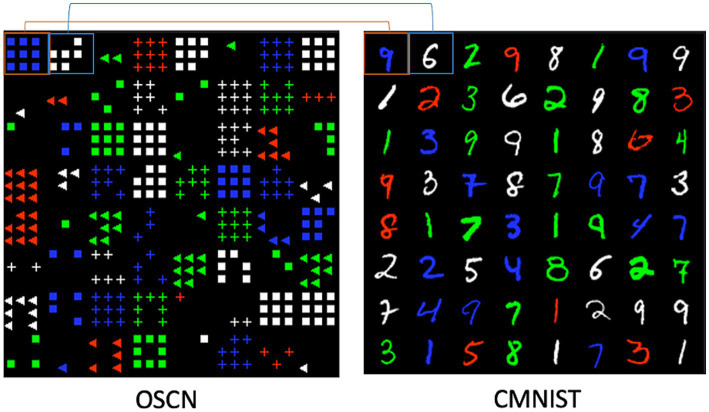
Sample data of the OSCN-CMNIST. The figure presents an 8 × 8 example of data from OSCN and CMNIST. Images at identical positions have identical numbers and colors to form pairs. For example, the top left image of OSCN is “9 blue squares” and that of CMNIST is “blue 9.” Likewise, the right image of OSCN is “6 white squares” and that of CMNIST is “white 6.”

To deal with the number sense, we used symbol and object images as the target modalities. These modalities differ from those involved in human sensory modalities such as vision, sound, or touch, and we refer to a modality as the mode and pattern of information following conventions in the machine learning domain. The concept of number can be represented using a symbol, such as “4,” or using object images, such as a picture of four squares (Figure 1). We created a dataset termed OSCN-CMNIST, which is a combination of the object-shape-color-number (OSCN) dataset and Colored Modified National Institute of Standards and Technology (CMNIST) database. Figure 2 presents sample data from OSCN-CMNIST. The OSCN refers to a set of synthetically created two-dimensional object images and represents the concept of numbers using these images. Each OSCN image comprises the following four factors: object layout, object color, object shape, and number of objects. CMNIST is a colored version of MNIST and is a commonly used dataset of handwritten digit images. The dataset represents the concept of numbers using symbolic Arabic numerals. An image pair was developed such that both images had identical numbers and colors. The shape and layout of the objects were created randomly. Detailed information is provided in Supplementary material.

### 2.2 Model

To model the cognitive processes required for the reconstruction and cross-modal generation tasks, we employed a mixture-of-experts multimodal variational autoencoder (MMVAE; Shi et al., 2019). The MMVAE is one of the generative models for multimodal learning that exhibits high performance in terms of generation quality. Furthermore, the MMVAE employs self-supervised learning, which does not rely on explicit teacher signals, similar to settings in the human developmental process. Although recent multimodal neural networks such as CLIP (Radford et al., 2021) exhibited outstanding performance, their information processing is complex. In contrast, MMVAE has the advantage of a straightforward neural network architecture, which is useful for discussing similarities with the brain. In addition, representations of the learned modalities are disentangled using shared and private latent space (details are provided later), making it easy to interpret the type of information encoded, and the way in which information is embedded.

**Figure 3:**
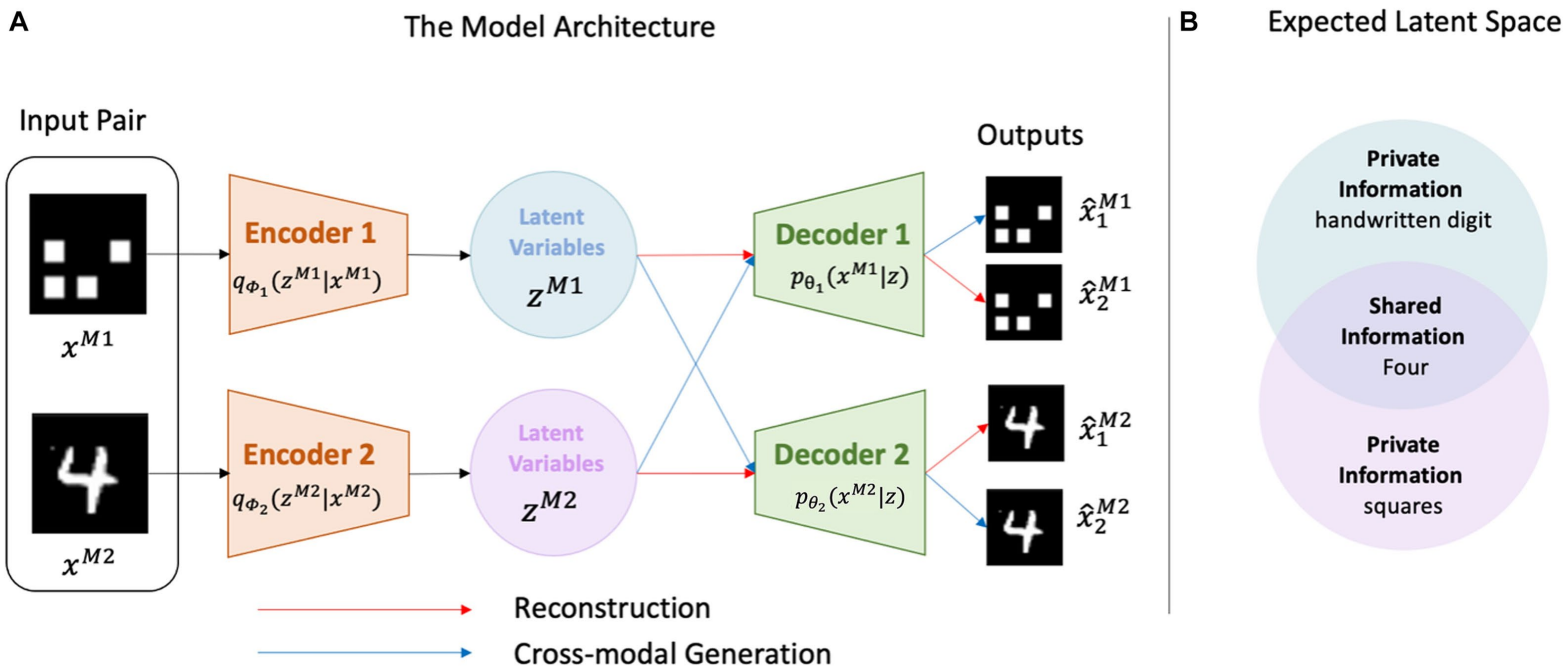
Overview of the model architecture. **(A)** The encoders produce latent variables using the input, whereas the decoders generate outputs from the latent variables. Each encoder/decoder corresponds to one modality. **(B)** The latent spaces are learned to include a subspace for the shared information, and the remaining spaces include private information.

Figure 3A illustrates the MMVAE architecture. For multimodal generation, pairs of data $\left(x^{M_{1}},\;x^{M_{2}}\right)$ were the inputs for model $A^{\text{multi}}$, which comprised two datasets with identical information but different modalities, namely $M_{1},M_{2}$. The encoders produced latent variables, namely $z^{M_{1}}$, $z^{M_{2}}$ and each decoder $D_{j}\left(j=1,\;2\right)$ generated outputs ${\hat{x}}_{i}^{M_{j}}$ for each latent variable $z^{M_{i}}\left(i=1,2\right)$. Collectively, the model simultaneously performed both reconstruction and cross-modal generation.

In artificial neural network models, because of training, information of input images (such as color, numbers, shapes, etc.) is embedded in the latent space of the network. In the case of MMVAE, the latent spaces may appear independent (Figure 3A); however, they could learn to overlap (Figure 3B). This is because the model is trained to output similar data regardless of the latent space from which the latent variable is sampled. Namely, in MMVAE, even when the modalities of input data differ, the same latent space is utilized. Therefore, it is expected that common information, such as color or numbers shared in both OSCN and CMNIST, utilizes a common mechanism in the latent space. On the other hand, in the case of the CMNIST modality, although it utilizes the same latent variable space as the OSCN modality, there is no need to leverage information such as the shape of the object (e.g., cross and triangle). Consequently, modality-specific (e.g., figure of Arabic numerals in the CMNIST and shape of objects in the OSCN dataset) information is represented by independent subspaces (private representation), whereas modality-general (e.g., color and number label) information is represented by a common subspace (shared representation).

We trained other models, namely $A_{1}^{\text{single}},\;A_{2}^{\text{single}}$(single-modal models), to learn the reconstruction only. Each $A_{i}^{\text{single}}$ model was assigned one $M_{i}$ modality and did not have access to other modalities, making it impossible for these models to learn the relationships among the modalities. Unlike in the multimodal model, the latent space did not spontaneously overlap. For these models, we ignored the blue arrows shown in Figure 3A. Decoder $D_{j}$ only used the corresponding latent variables $z_{j}$ to output ${\hat{x}}_{j}^{Mj}$, and the two variational autoencoders were trained independently.

Following training, the multi-model $A^{\text{multi}}$ learned latent representations for both $M_{1},\;M_{2}$ modalities, whereas the single-modal models $A_{1}^{\text{single}}$, $A_{2}^{\text{single}}$ only learned a latent representation for the corresponding modality $M_{i}$. Both models learned the latent representations of $M_{i}$, but in different ways. For training, MMVAE maximizes the following objective function (Shi et al., 2019):


$$L^{MoE}\left(x^{M_{1}},\;x^{M_{2}}\right)=\frac{1}{N_{M}}\sum_{m=1}^{N_{M}}E_{z∼q_{\phi }\left(z\;|\;x^{M1:M2}\right)}\left[\log\frac{p_{\Theta }\left(z,x^{M_{1}:M_{2}}\right)}{q_{\phi }\left(z\;|\;x^{M_{1}:M_{2}}\right)}\right]$$

where $N_{M}$ denotes the number of modalities, z is the latent variable, and x is the input data. Intuitively, the objective function denotes the average of the evidence lower bounds for each generation path. Please refer to Supplementary material for additional details regarding the implementation.

## 3 Results

### 3.1 Output

**Figure 4:**
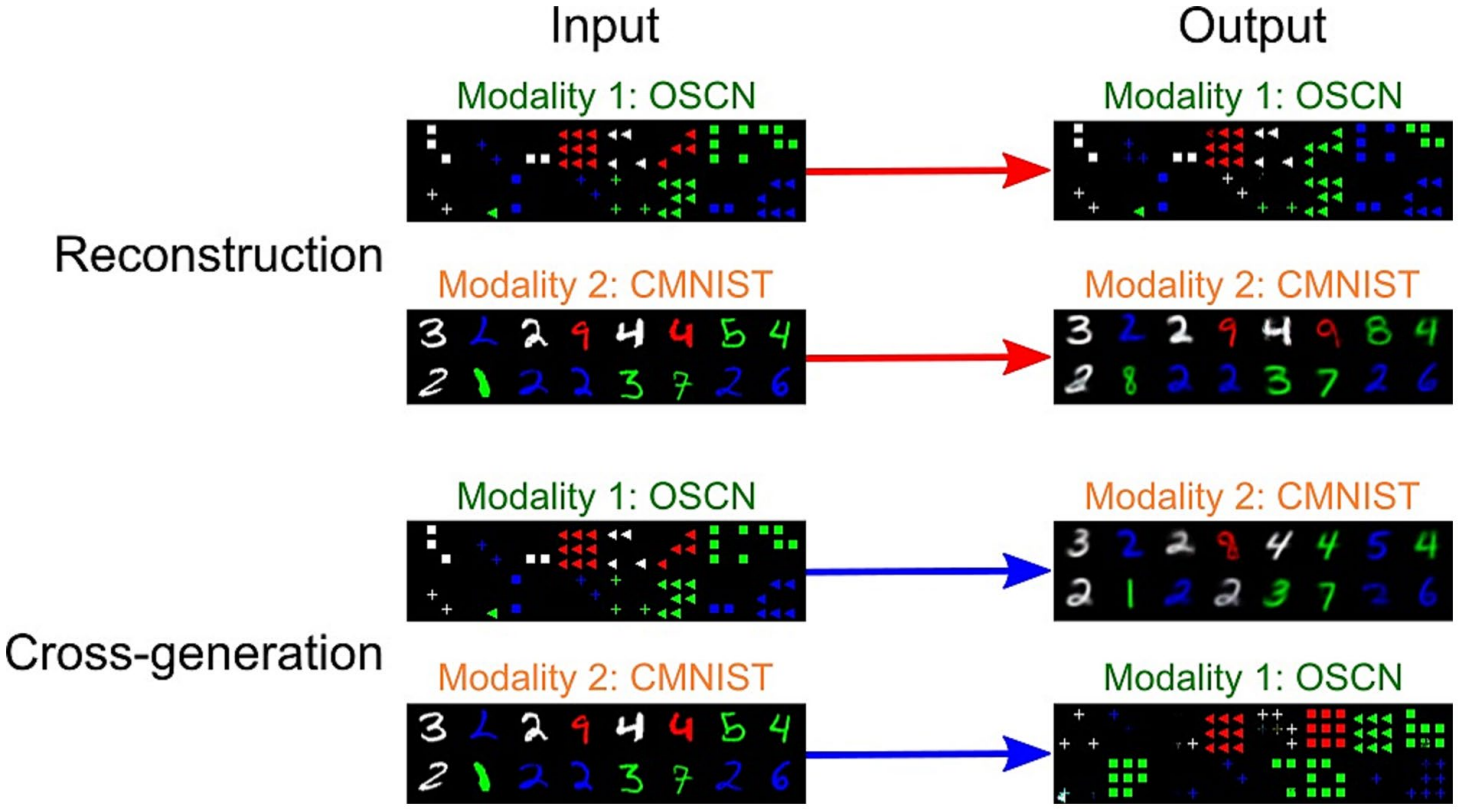
Sample inputs/outputs from the trained multimodal model. The images at identical positions correspond to each other.

Figure 4 depicts the outputs of the multimodal models trained using OSCN-CMNIST, including the reconstructed and cross-generated images (images generated from other modalities). The tasks were successfully learned in both modalities, as the output images were clear and precise; however, the cross-modal generated output included some errors when inputting CMNIST images.

**Figure 5:**
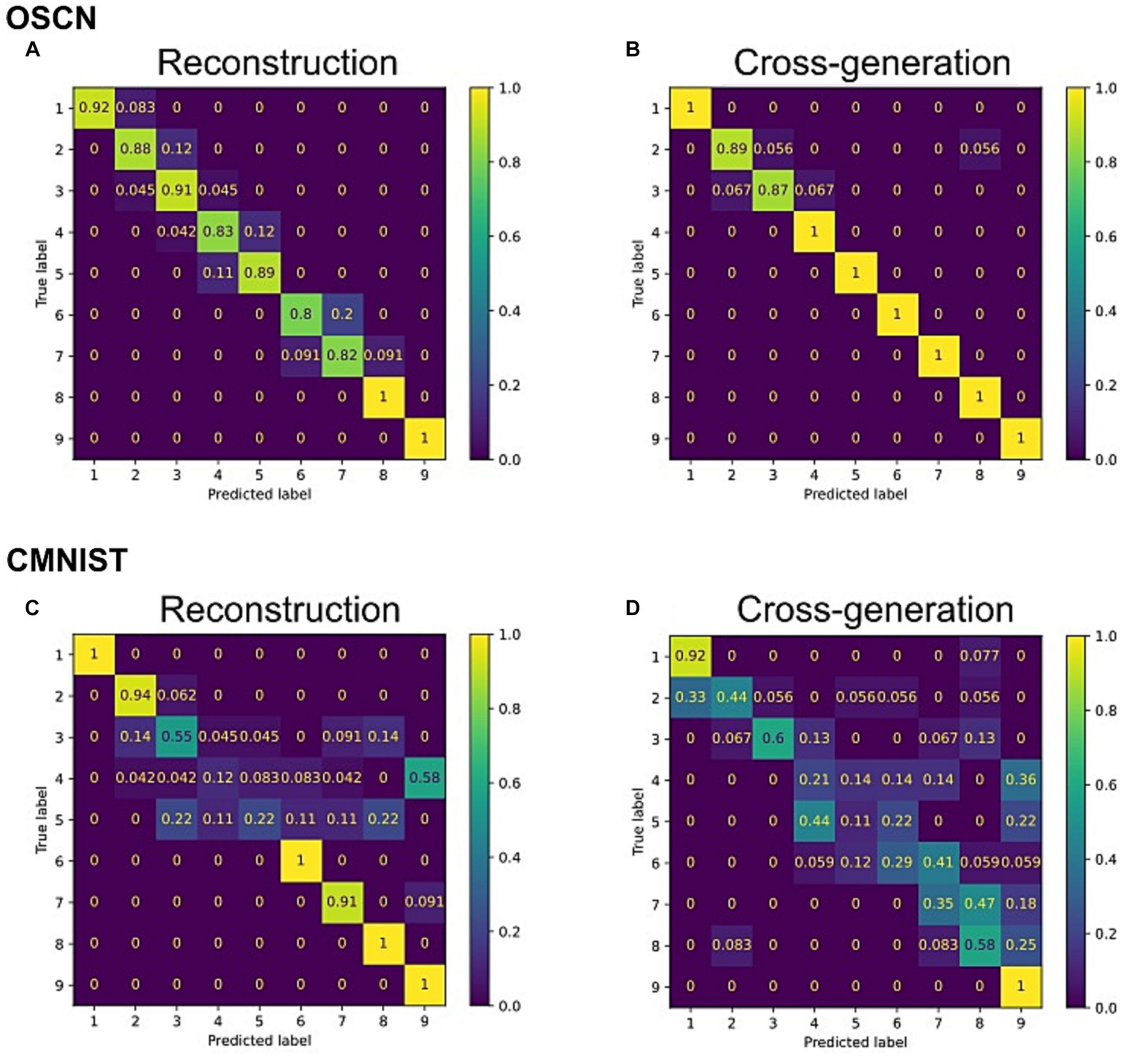
Confusion matrix between true number label and outputs from the trained multimodal model. **(A)** The reconstruction task for the OSCN images. **(B)** The cross-generation task to the CMNIST from OSCN images. **(C)** The reconstruction task for CMNIST images. **(D)** The cross-generation task to the OSCN from CMNIST images.

To quantitatively analyze the performance of the model, we trained additional neural network models to determine to which class (e.g., number) the output of the multimodal model corresponded to. We visualized the agreement rate between true labels, i.e., the number represented by original images before reconstructing and cross-generating, and predicted labels by the classifier model using reconstructed and cross-generated images (Figure 5). In both CMNIST and OSCN datasets, regardless of input numbers, most accuracies of reconstructions and cross-generation were above chance levels (1/9 = 0.111).

We repeated the same procedure and conducted statistical tests on the generation ability of 20 distinct networks. The results showed that the accuracy of the multimodal model outperformed significantly chance levels in reconstruction of the OSCN and CMNIST datasets [*t*(19) = 6.69, *p* < 0.0001 in the OSCN dataset and *t*(19) = 6.72, *p* < 0.0001 in the CMNIST dataset using one-sample *t*-test]. The accuracy of cross-generated images significantly outperformed chance levels when the OSCN dataset was used as inputs [*t*(19) = 7.19, *p* < 0.0001]. Similarly, when the CMNIST dataset was used as inputs, the multi-modal model performed the cross-generation task above chance levels [*t*(19) = 6.66, *p* < 0.0001].

### 3.2 Qualitative analysis of latent representation

**Figure 6:**
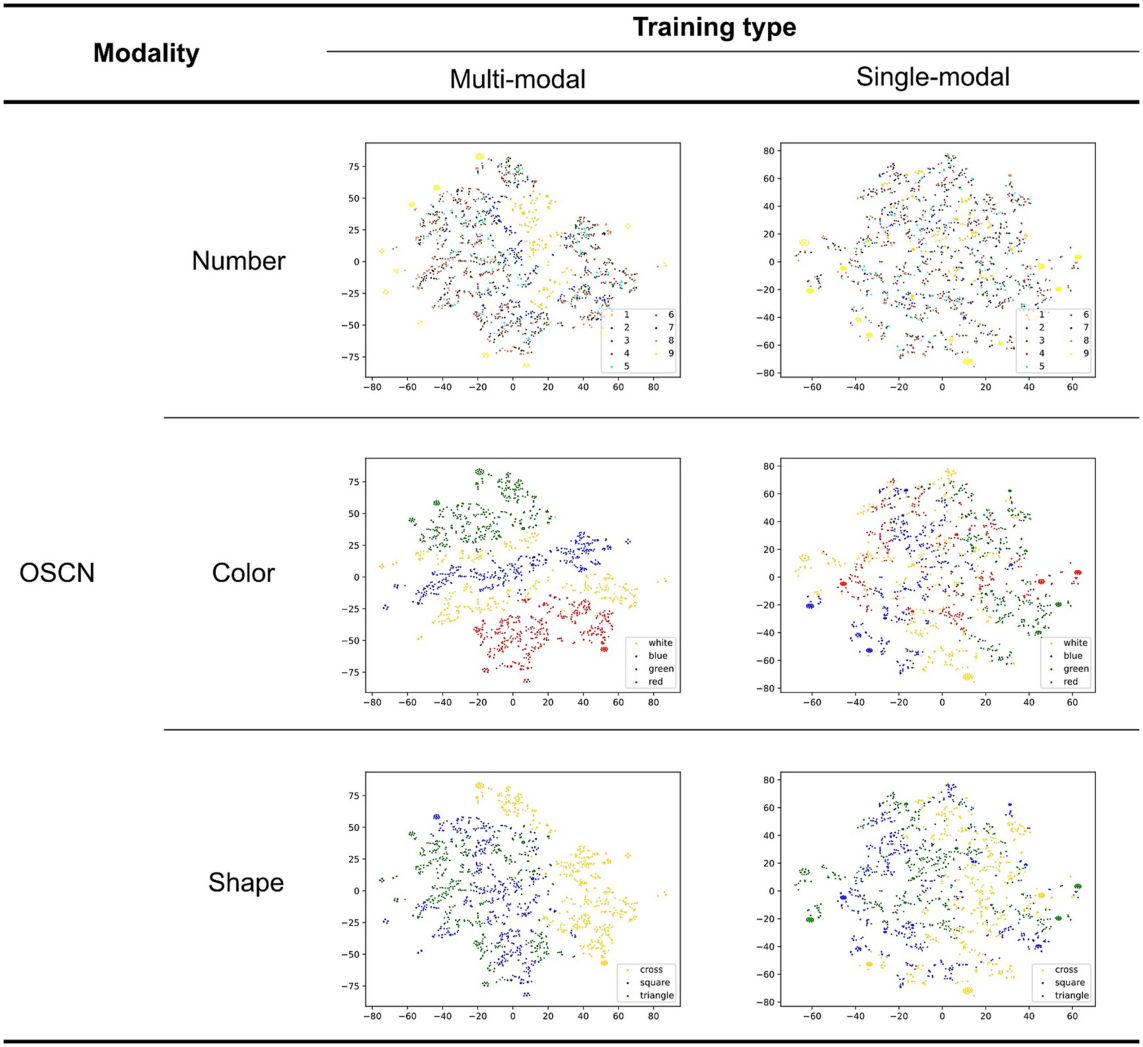
Two-dimensional latent representation of the OSCN with different training types. Different color points belong to various number/color/shape classes in the first, second, and third rows, respectively.

Figure 6 illustrates the latent representations of the OSCN generated by the models using different training methods. We provided test data of each modality to each model to visualize the latent space. The model’s encoder generated the latent variables using the input. These variables were transformed using t-distributed stochastic neighbor embedding (t-SNE; van der Maaten and Hinton, 2008) to reduce the number of dimensions from 20 (original dimension of the latent space) to two. In each learning method, all rows depict the identical latent representations, as they were estimated using the same images. However, different colors were assigned to each point (the upper, middle, and lower rows represent the number, color, and figure classes, respectively).

The quality of the latent representations of the numbers appears to improve in the multimodal model. This is because some clusters became more cohesive and the number of mixed points (points in the wrong cluster) decreased. Moreover, we identify an order based on the magnitude of the corresponding class. By using numerical order, the neural network model can discern size relationships, such as larger and smaller. Because of this, the model successfully learned to judge the magnitude of the numbers.

The single-modal model produced a well-clustered latent representation for shapes, although there was a mixing of different clusters for color and number classes. In contrast, the multimodal model appeared to mainly cluster points based on their color in addition to shape (identical points of similar color were adjacent). The increased focus on color classes may be attributed to the shared color modality between OSCN and CMNIST.

In summary, the multimodal model generated more divided clusters for the number, with the order based on the class magnitude. The clustering quality for shapes was similar to that of the single-modal model; however, it focused more on color.

**Figure 7:**
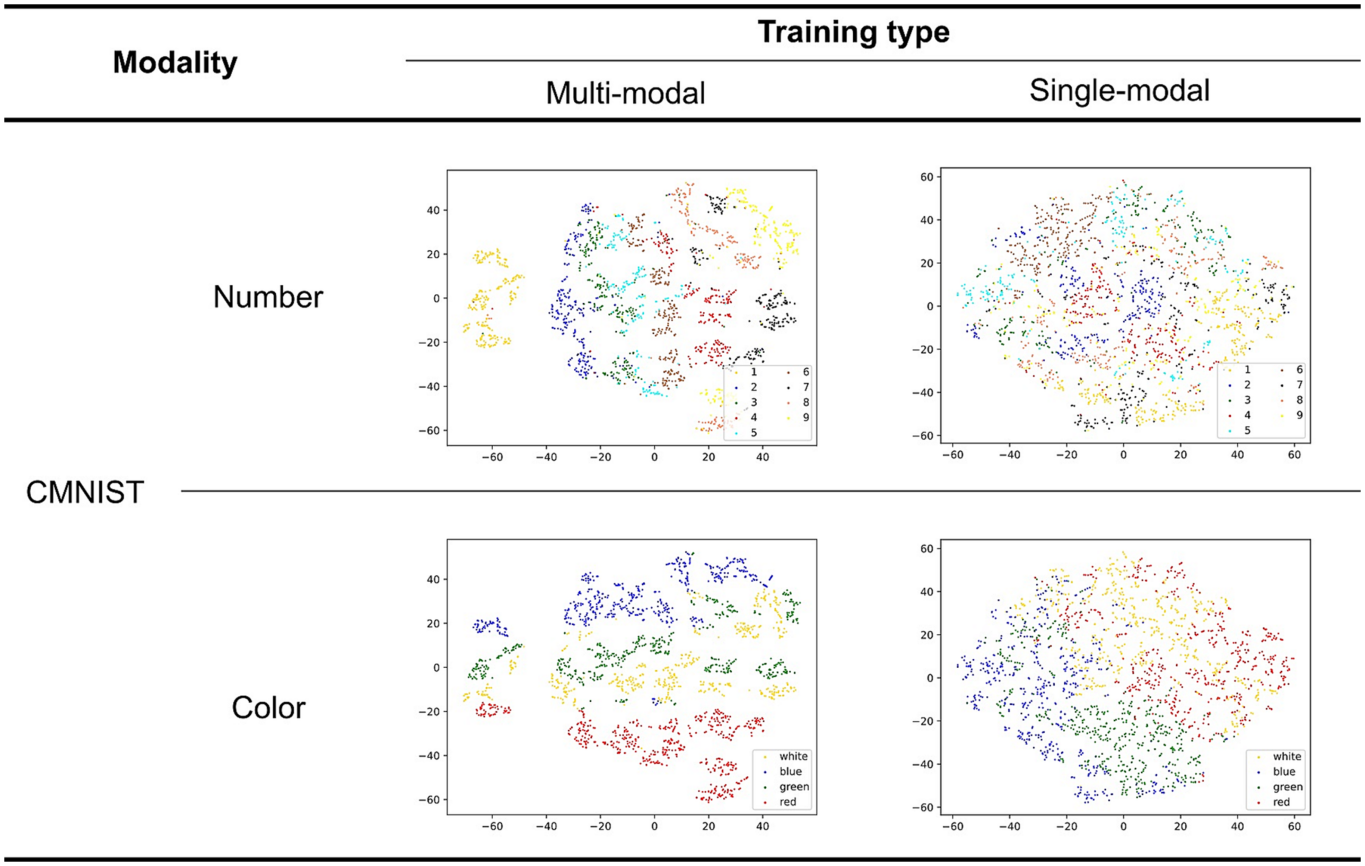
Two-dimensional latent representation of the CMNIST with different training types. The upper and lower row visualizations are based on the number and color classes, respectively. Different color points belong to dissimilar numbers/color classes in the first and second rows.

Figure 7 shows the latent representations of CMNIST generated in a manner similar to that shown in Figure 6. All rows displayed a similar representation; however, different colors were assigned to each point (the upper and lower rows visualize the number and color classes, respectively).

The quality of the latent representations of numbers improved in the multimodal learning model. This is supported by the fact that points belonging to the same class are not mixed in the case of the multimodal learning model. Additionally, an order based on the magnitude of the corresponding class supposedly existed in a multimodal manner as observed in the OSCN representation. This was the result of multimodal learning, in which the model attempted to associate the two modalities. This order was not observed in the representation generated by the single-modal model because it did not include magnitude information. Although mixtures were observed in the number class (e.g., 7 and 9, as well as 3 and 5, were positioned closer to each other in the latent space of the multimodal model), this can be explained by the similarity in the shapes of Arabic numerals in the CMNIST images. Furthermore, the multimodal model successfully clustered the points based on color than the single-modal model. This is because OSCN and CMNIST possess common colors, and the multimodal model learned the colors to gain a shared representation.

In summary, the multimodal model clustered the data based on shape and color, and simultaneously ordered points based on the magnitude of the number.

### 3.3 Quantitative analysis of latent representation

From the qualitative analysis, the multimodal model appeared to modify the clustering structure and learn better number sense. To confirm this observation, we introduced a silhouette coefficient to qualitatively measure the clustering value. The distance between different clusters increased as the value approached 1, concomitant with a decrease in the distance between points in similar clusters. We calculated the silhouette coefficient using the latent values. Notably, the dimension reduction algorithm did not affect the results because the silhouette coefficient was calculated in the original latent space. Additional calculation details are provided in Supplementary material.

**Figure 8:**
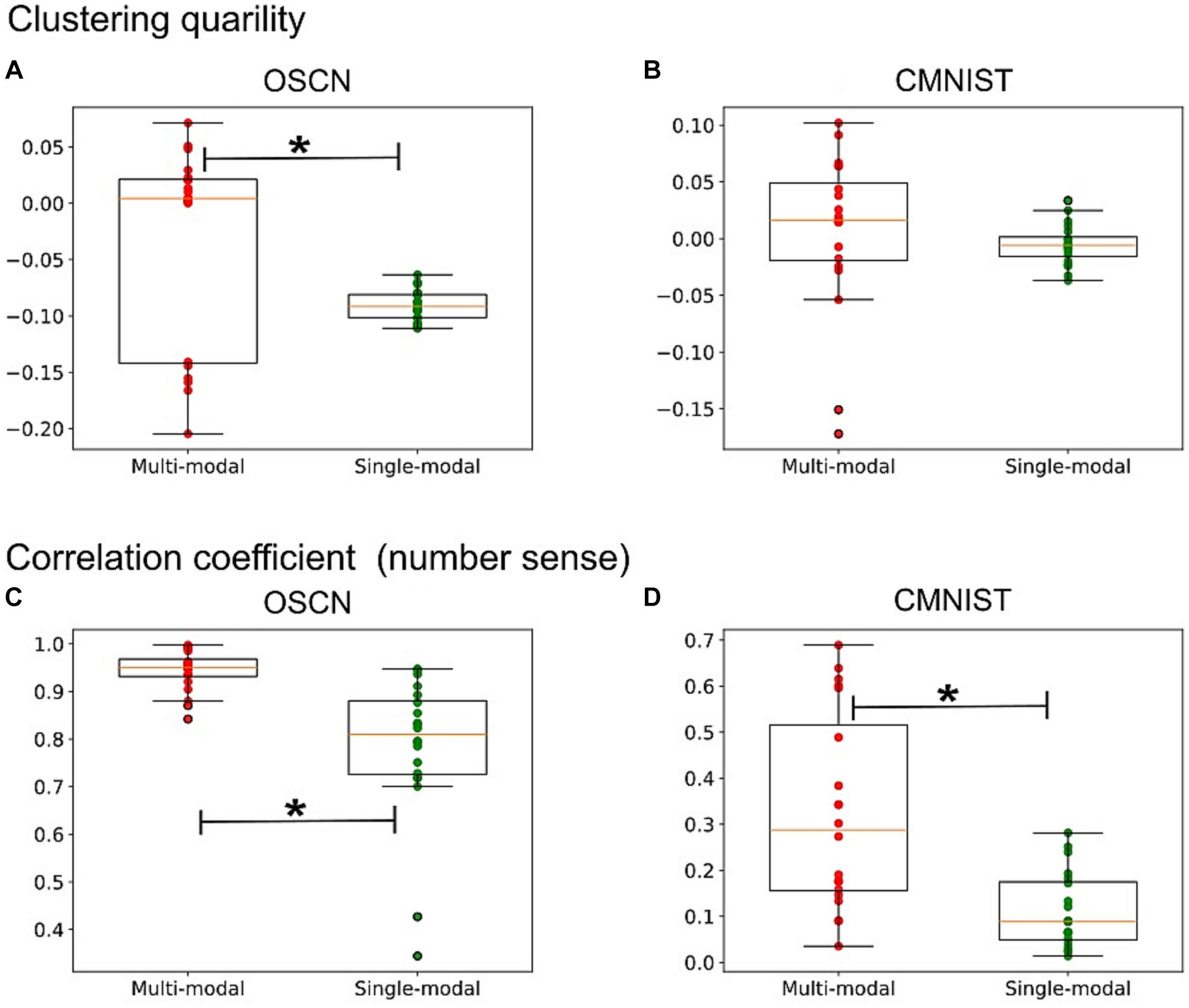
The quantitative analysis of latent representations. **(A,B)** A comparison of the silhouette coefficients for the latent representations learned in different ways in the OSCN **(A)** and CMNIST **(B)** datasets. **(C,D)** A comparison of the correlation coefficient between the latent distance (e.g., the distance between the average of points in the latent space belonging to “2” and “5”) and the class distance (e.g., the class distance between “2” and “5” is “3”) in the OSCN **(C)** and CMNIST **(D)** datasets. ^*^p < 0.05.

Figure 8 (upper) shows a comparison of the silhouette coefficients for each representation. For both modalities, the silhouette coefficient was higher when the models were learned in a multimodal manner. The statistical test revealed the superiority of multimodal model in the OSCN dataset [*t*(19.92) = 2.84; *p* = 0.0102], but not in the CMNIST dataset [*t*(21.46) = 0.71; *p* = 0.4826]. Therefore, multimodal learning has potential to improve clustering quality.

We attempted to quantitatively measure the quality of the learning of number sense. Upon learning a numerical quantity, the distance between clusters $C_{i}$, $C_{j}$ (defined as the distance between the average of the points in one cluster and that of the other cluster) is expected to be associated with the difference between the corresponding two class numbers i, j, that is $\left|i-j\right|$. For example, the difference between Clusters 1 and 3 should be greater than that between Clusters 1 and 2. In such cases, the cluster distance (distance between clusters in the latent representation) and class distance (distance between corresponding classes) should exhibit monotonically increasing relationships.

Figure 8 (lower) shows the correlation coefficient between the cluster distance and class difference. A value close to one indicates that the model successfully learned the magnitude relationship between numbers (and the number sense). The correlation coefficient increased after the modality was learned in a multimodal manner. In particular, the CMNIST representation displayed a remarkable change [*t*(24.57) = 4.18; *p* = 0.0003 in the CMNIST dataset]. Additionally, the coefficient of the multimodal model for the OSCN outperformed that of the single-modal learning model [*t*(21.83) = 4.51; *p* = 0.0002 in the OSCN dataset].

### 3.4 Downstream arithmetic task

**Figure 9:**
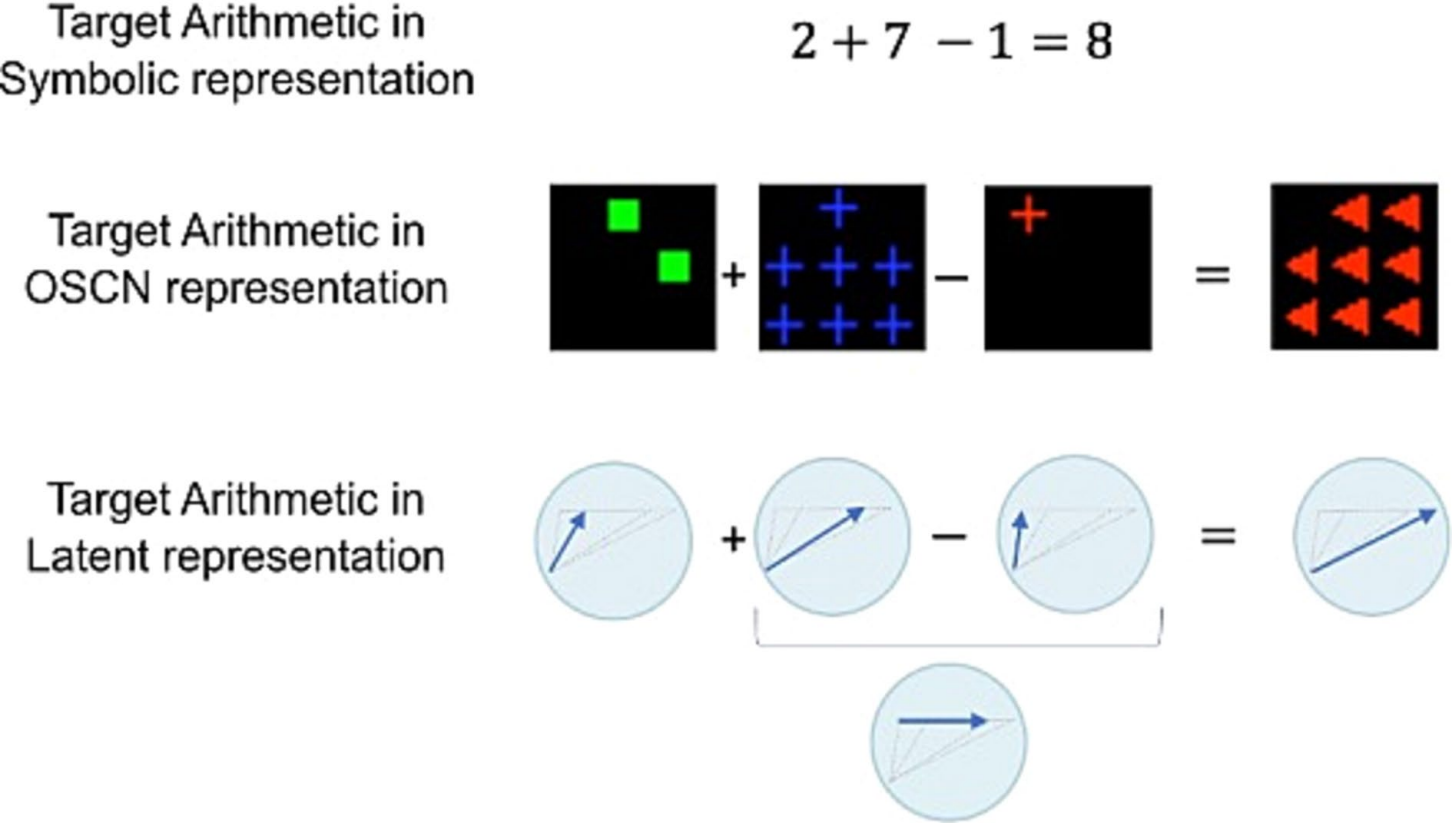
Arithmetic task overview. Identical arithmetic operations can be represented in various ways. If the model successfully learns the quantitative sense, it can perform additions and subtractions.

Multimodal learning was effective for obtaining a sophisticated representation of data in terms of clustering and number sense. This warrants investigation into the usefulness of latent representations for downstream cognitive tasks that require number sensing. Therefore, the models were tested using arithmetic tasks. Figure 9 presents an overview of this task. For a learned latent representation that reflects the quantitative relationship between data, the model may perform addition and subtraction using the representation.

**Figure 10:**
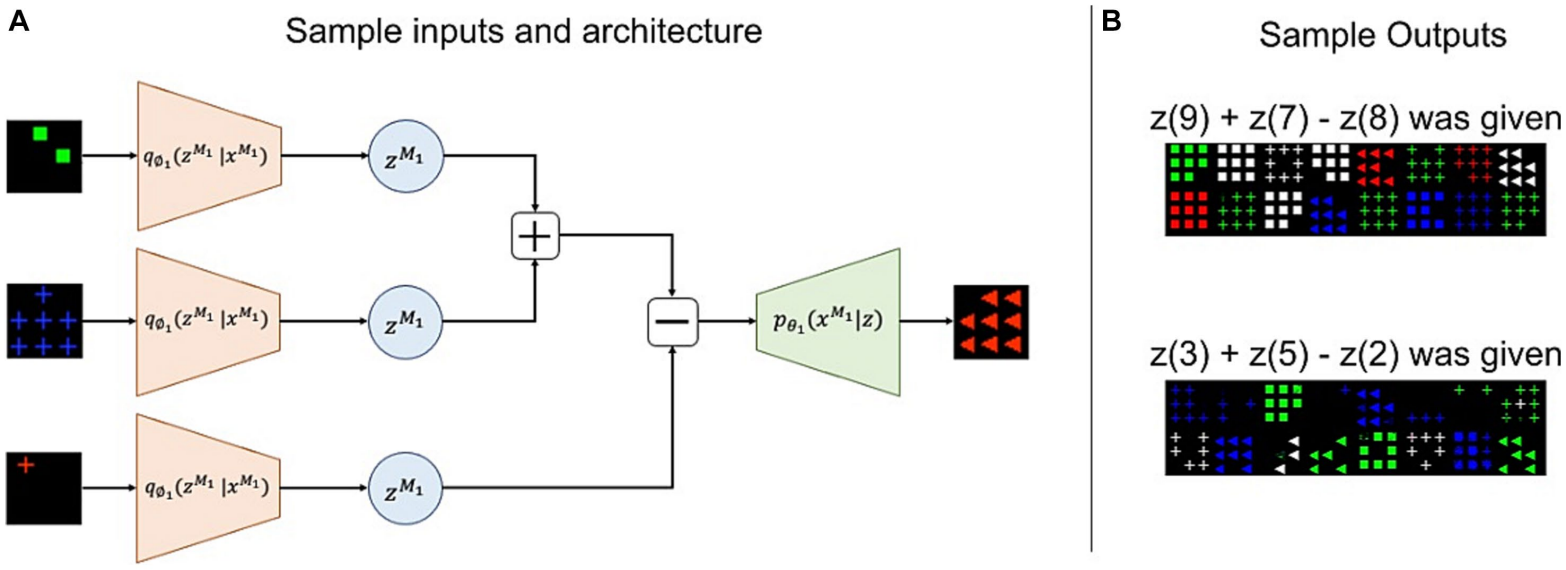
Sample inputs/outputs and architecture for the arithmetic task. **(A)** Three data inputs are provided to the model. The model generates latent variables using the input and performed addition and subtraction in the latent space. Subsequently, the calculation result was used to produce the final output. **(B)** Actual outputs upon executing “9 + 7 − 8” and “3 + 5 − 2” using OSCN. “8” and “6” are expected to be produced.

Figure 10A depicts the architecture of this task using sample inputs. The OSCN images of 2, 7, and 1 provided to the corresponding encoder produced latent variables $z\left(2\right),\;z\left(7\right),\;\text{and}\;z\left(1\right)$, respectively (assuming $z\left(i\right)$ to be a latent representation of the OSCN images of i). Subsequently, we performed addition and subtraction using these latent variables outside of the neural network and provided the results to the decoder $D_{\text{OSCN}}$ to generate images as follows: $D_{\text{OSCN}}\left(z\left(2\right)+z\left(7\right)-z\left(1\right)\right)$ where $D\left(z\right)$ denotes an image created by D for a certain z. If $z\left(2\right)+z\left(7\right)-z\left(1\right)$ is close to $z\left(8\right)$, the final output image would appear like the OSCN image of 8. This is because $D_{\text{OSCN}}$ is trained such that $D_{\text{OSCN}}\left(z\left(i\right)\right)$ and the OSCN image of i are identical in appearance. The models were not trained for this task, and we used only the latent representations obtained through generation training. Figure 10B depicts the actual output when the $z\left(9\right)+z\left(7\right)-z\left(8\right)$ and the $z\left(3\right)+z\left(5\right)-z\left(2\right)$ was provided to $D_{\text{OSCN}}$. Despite some errors, it produced several images depicting the numbers “8” and “6.”

To compare the performances, we assessed several arithmetic expressions using different models and counted the number of accurate images. The arithmetic expressions were selected to cover various input and answer patterns (are shown in Supplementary material).

**Figure 11:**
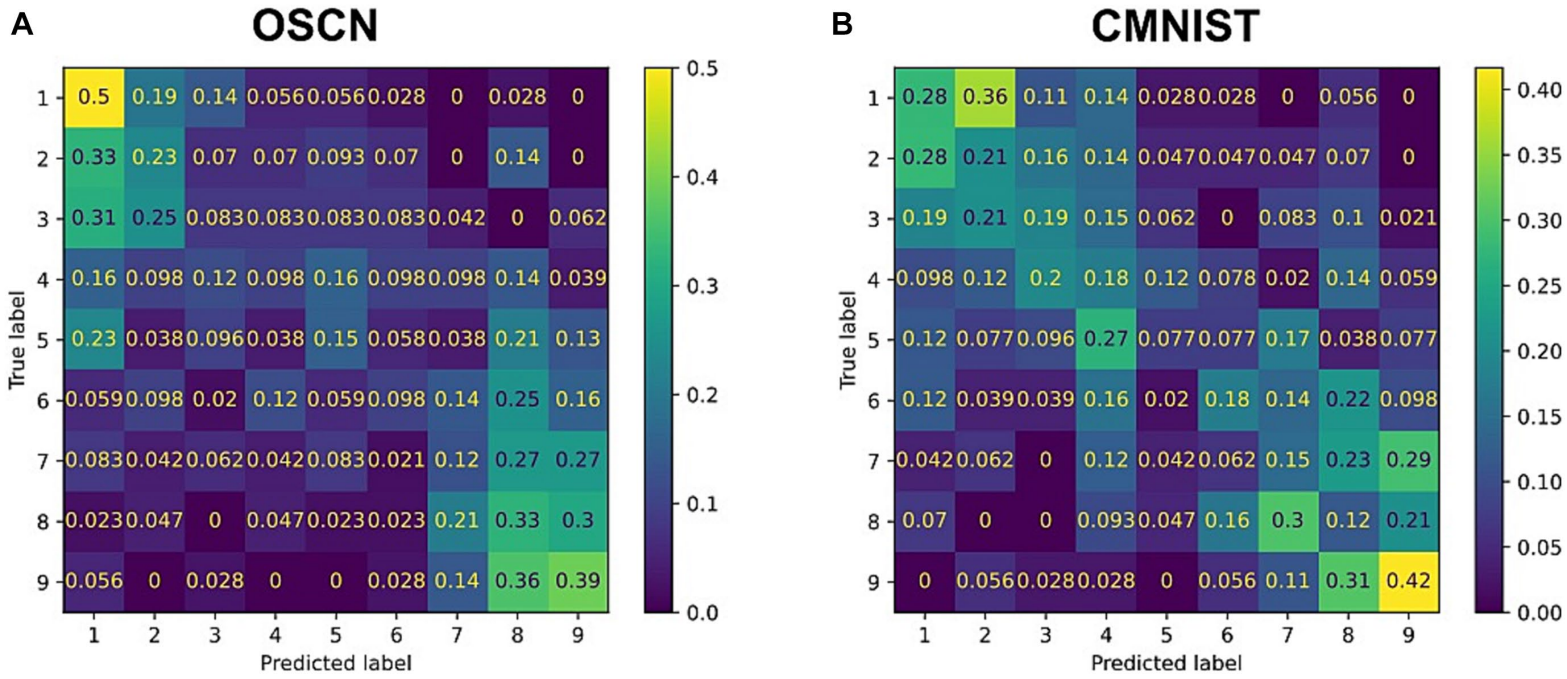
Confusion matrix between true answers and generations by multimodal learning model. The results of the arithmetic task when inputting the OSCN **(A)** and CMNIST **(B)** images. The y-axis numbers represent true answers of arithmetic calculation while the x-axis numbers represent predictions made by classifiers that were given generated images by multimodal models.

Figure 11 represents the accuracies estimated by the same classifier used in the “output” section. In Figure 11, the y-axis numbers represent true answers of arithmetic calculation while the x-axis numbers represent predictions made by classifiers that were given generated images by multimodal models. As shown in Figure 11, the multi-modal model outperformed chance levels (1/9 = 0.111) in most cases. Confusions were observed when the true answers had a moderate magnitude, such as 3, 4, and 6, in the OSCN dataset. In some cases, even when predictions were wrong, the answers by the neural network were close to the true label, e.g., when the true label is “5,” the “4” prediction (27%) is higher than the “9” prediction (7.7%) in the CMNIST dataset.

**Figure 12:**
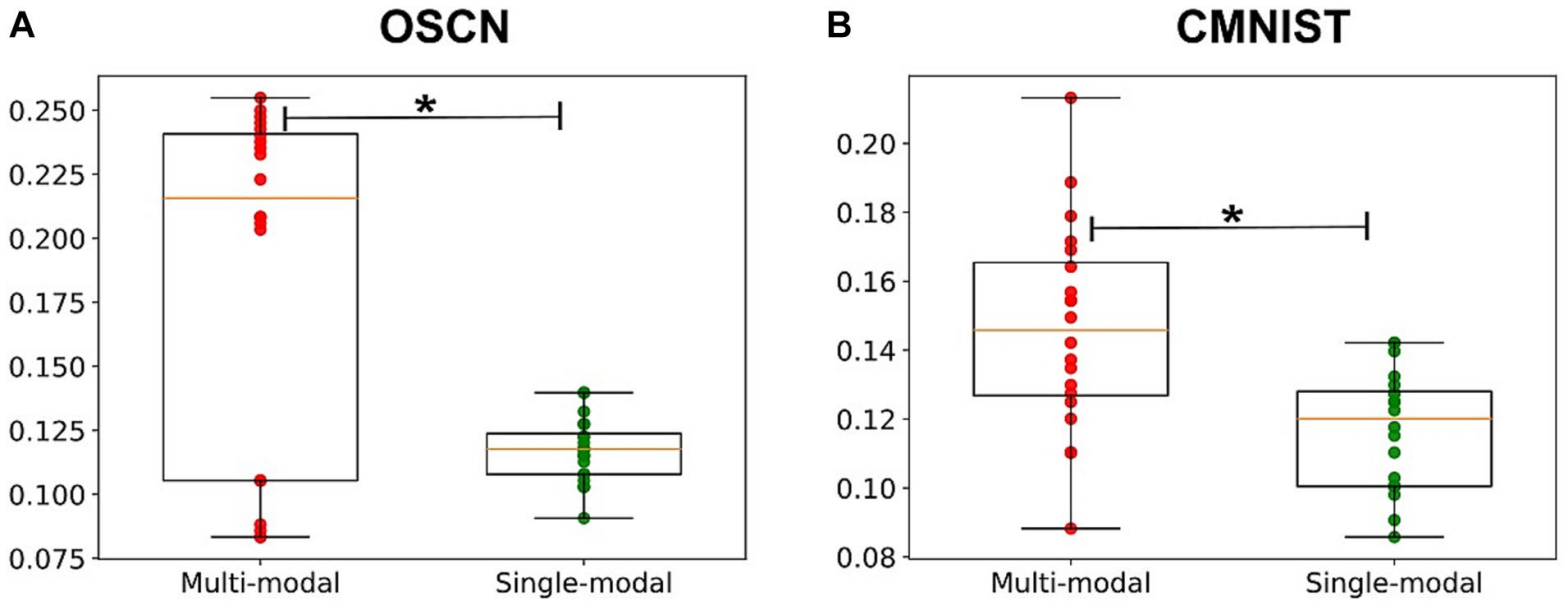
Comparison of the success rate of the arithmetic task. The success rate of the arithmetic task when inputting the OSCN **(A)** and CMNIST **(B)** images. ^*^p < 0.05.

Figure 12 compares the success rates of each model. The multimodal model displayed better performance in both datasets [*t*(30.35) = 3.82; *p* = 0.0006 in the CMNIST dataset and *t*(20.31) = 4.71; *p* = 0.00013 in the OSCN dataset]. Through multimodal learning, numerosity, which was originally absent in the CMNIST modality was embedded in the neural network model.

## 4 Discussion

In this study, we trained models in single and multimodal manners to compare the latent representations of symbolic numbers and object images. Multimodal training produced better latent representation in terms of clustering quality, which is consistent with previous findings on multimodal learning in deep neural networks (Suzuki et al., 2016; Guo et al., 2019). A better latent representation in multimodal learning is intuitively understandable because learning can access more diverse information during training; however, the underlying mechanisms remain unclear. Shared representation may be an important factor (Ngiam et al., 2011). In the current experiment, the multimodal model obtained a shared representation for color, which was a shared factor in the training dataset. The model successfully identified an association between the two datasets, whereas the single modal model did not learn in a similar manner (see Supplementary Figure S2 for a visual explanation). The number was another shared modality learned together in the multimodal model. Therefore, the multimodal representation of CMNIST reflects the number sense learned in the OSCN and shared across modalities. The representation of the number sense in CMNIST is not trivial. This is because CMNIST does not contain information on the magnitude of the numbers, unlike OSCN, which contains the number of objects as images. The single-modal representation of CMNIST does not reflect number sense. However, number sense was better obtained with the combined learning of OSCN and CMNIST. This may be attributed to the use of CMNIST as a label during training, which facilitated the assembly of OSCN images by the presented number. Moreover, the representation learned by the multimodal model exhibited superior performance in downstream arithmetic tasks. Therefore, multimodal learning facilitated the learning of an improved representation of information and performed tasks using the target information.

In the field of machine learning, multimodal learning leads to more disentangled representations by visualizing latent representations (Suzuki et al., 2016; Zhou and Shen, 2020). Our results support these findings and highlight the following perspectives: (1) quantitative evaluation of the learned representations and (2) the contribution of changed representations for better performance of the downstream tasks. These findings reveal the fundamental advantages of multimodal learning, which were not considered in previous studies.

Moreover, our study introduced multimodal learning in the context of the computational modeling of cognitive abilities, particularly the acquisition of mathematical ability. With regard to mathematical skills, the exact process by which the human brain grasps the concept of symbolic numbers remains unclear (Diester and Nieder, 2007; Nieder and Dehaene, 2009; Testolin, 2020). Previous studies have reported the acquisition of number sense using computational models, for example, spatial filter (Park and Huber, 2022; Paul et al., 2022), untrained neural networks (Kim et al., 2021; Lee et al., 2023), neural networks trained on object recognition tasks not limited to number images (Nasr et al., 2019; Nasr and Nieder, 2021), and neural networks trained with number images (Stoianov and Zorzi, 2012; Testolin et al., 2020; Mistry et al., 2023). These studies commonly have indicated that acquiring number sense is possible using only single-modal information. Although we emphasized the importance of the MMA, our findings are not inconsistent with this hypothesis in the sense that number sense could self-organize without explicit instructions, as observed in the single-modal setting (OSCN in Figures 6, 8). Other research demonstrated that without the explicit use of multimodal information, the transformer model can acquire a well-structured latent representation similar to the mental number line, in which addition and subtraction calculations can be performed (Boccato et al., 2021). Our study distinctly showed that integrating symbolic numbers with object information not only facilitates structured latent representations but also significantly enhances the performance of downstream arithmetic tasks, including addition and subtraction beyond single-modal learning. Similar to our study, several studies discussed arithmetic abilities using neural networks that process multimodal information (Verguts and Fias, 2004; Di Nuovo and McClelland, 2019; Sabathiel et al., 2020). In particular, Verguts and Fias (2004) is one of the few exceptions that studies the representation of numbers by providing symbolic and non-symbolic information to neural network models; however, their study is limited in that they adapt a hard-coded model and use symbolic numbers as one-hot vectors. In contrast, in our study, the CMNIST images were handwritten, and the various visual patterns represented the same number of symbols; therefore, the neural network must extract latent information from the symbol and object images. In this respect, our study is similar to the multimodal setting in a real-world environment.

In addition to cognitive ability, studies on multimodal learning in the context of machine learning may contribute to an understanding of the mechanisms of neuropsychological symptoms. Synesthesia is an illustrative example of altered MMA, a phenomenon in which one sensory stimulation evokes other senses simultaneously, such as observing a color on a certain character (Hubbard and Ramachandran, 2005). Several studies have attempted to computationally model synesthesia (Makioka, 2009; Yamaguchi et al., 2013). Our findings may provide substantial insights into the understanding of synesthesia as an alteration in shared and private latent representations. In addition, we found that multimodal representation contributed to enhanced performance of arithmetic tasks. This result is consistent with those of previous clinical studies suggesting that persons with synesthesia possess advantages regarding cognitive performance related to space/time recognition and memory (Kadosh et al., 2011; Ovalle-Fresa et al., 2021). As another example of neuropsychological symptoms, we expect that altered latent representations in the MMA may explain the peculiar phenomenon of savant syndrome, a rare condition in which patients with mental disorders exhibit superior talents in specific domains, such as memory, mathematics, and the arts (Treffert, 2009). For example, the astonishing skill of calendar calculation, one of the representative talents of savants, can be understood as a unique latent representation acquired through extreme MMA, with the modalities of spatial arrangements of numbers in calendar sheets and symbolic information of days of the week. This hypothesis is supported by previous studies (Bouvet et al., 2014; Hughes et al., 2019), thereby suggesting an association between synesthesia and savants. Our simulation provides a computational explanation for this hypothesis. Additional modeling studies using the simulated lesion method in computational psychiatry (Yamashita and Tani, 2012; Idei et al., 2021) may lead to a formal computational understanding of synesthesia/savant syndrome.

However, this study has some limitations. The CMNIST-OSCN dataset is relatively simple because it was created using a synthetic dataset. In addition, only modalities with vision information were used as multimodal information. To overcome these limitations, future studies should use more diverse sensory modalities and conduct experiments using large-scale real-world data. Given the simplicity of the dataset, we cannot exclude the possibility that the network gauges numerosity based on the total area and convex hull rather than the actual count of objects. To address this potential confounding factor, future studies should consider controlling such low-level visual features by drawing on the methodology of previous computational simulation studies (Nasr et al., 2019; Testolin et al., 2020). Nevertheless, even if the MMVAE relies on these low-level visual features, its capability to extract physical magnitude from the OSCN and correlate the acquired representations of physical magnitude with CMNIST remains a consistent finding.

The dimension reduction algorithm may influence the visualization and qualitative results. Although a quantitative analysis of the latent variables was conducted before dimension reduction, we could have proposed an alternative hypothesis if different visualization algorithms had been used. Related to this, the size effect, which indicates that the mental distance between numbers is not linear to the distance between the magnitudes of these numbers (Nieder and Miller, 2003), was not observed in the compressed space. Indeed, previous studies have suggested that numerosity is encoded in neurons using logarithmic transformations rather than linear ones (Nieder and Miller, 2003; Stoianov and Zorzi, 2012; Nasr et al., 2019). We conducted additional analyses based on the hypothesis that the latent space before dimensionality reduction encodes numerosity using a nonlinear scale (section 4 in Supplementary material). The results showed a stronger correlation between latent representations and numerosity under the assumption of linear relationships, rather than nonlinear relationships. Behind the discrepancy with previous studies, there may lay differences in model architecture and input stimulus. Interestingly, Verguts and Fias (2004) did not clearly find nonlinear representation when artificial neural networks processed both symbols and non-symbols. This indicates that linear representations may be preferred in advanced cognitive processes that involve symbols.

Furthermore, downstream arithmetic tasks, such as addition and subtraction, are basic compared with human mathematical skills. Future studies should also include more complicated downstream tasks. For example, algebraic operations such as addition and subtraction are not learned by the neural network model because the latent state values obtained by inputting MINST or OSCN images were added or subtracted outside of the neural network. In future research, it will be essential to model how the neural system acquires algebraic operations. Incorporating insights from human neural representations of algebraic operations (Nakai and Nishimoto, 2023) may prove beneficial in the modeling process. The successful replication of more complicated cognitive skills should enable a comparison between the human brain and neural models. This, in turn, could reveal the detailed correspondence between the computational mechanisms in multimodal models and biological phenomena in the human brain. By addressing these challenges, a computational approach using artificial neural networks, as in our research, has the potential to offer comprehensive insights into the cognitive and neuroscientific mechanisms underlying MMA.

## Data availability statement

The data presented in the study are deposited in the Github repository, which could be accessed via https://github.com/ncnp-cpsy/NumberSenseAndMultimodalLearning.

## Author contributions

KN: Conceptualization, Formal analysis, Investigation, Methodology, Resources, Software, Validation, Visualization, Writing – original draft, Writing – review & editing, Data curation. TS: Conceptualization, Investigation, Methodology, Resources, Software, Validation, Visualization, Writing – review & editing. YY: Conceptualization, Funding acquisition, Investigation, Methodology, Project administration, Resources, Supervision, Validation, Writing – review & editing.
