# Supplementary material for: Emergence of number sense through the integration of multimodal information: developmental learning insights from neural network models
Source: Front Neurosci. 2024 Jan 17;18:1330512. doi: 10.3389/fnins.2024.1330512 (PMC10828047; doi:10.3389/fnins.2024.1330512)
Supplement: Supplementary file 1 [file Data_Sheet_1.PDF]

Supplementary Material

1 Dataset

1.1 CMNIST

We created CMNIST, a colored version of MNIST, by assigning one color (out of the four colors: blue, white, green, and red) to MNIST images. CMNIST consists of 120,000 training data and 4,000 test data images. The number of images for each color and numerical class in the dataset is presented in Tables S1 and S2.

Table S1. The number of images for each color class in the CMNIST dataset.

	**Blue**	**White**	**Green**	**Red**
**Training**	30,000	30,000	30,000	30,000
**Test**	1,000	1,000	1,000	1,000

Table S2. The number of images for each numerical class in the CMNIST dataset.

	**1**	**2**	**3**	**4**	**5**	**6**	**7**	**8**	**9**
**Training**	15,200	13,092	13,588	12,940	12,028	13,188	13,780	12,796	13,388
**Test**	556	508	460	488	388	372	420	388	420

1.2 OSCN

The number of images for each shape class in the OSCN dataset are shown in Table S3. The numbers of images for each color and numerical class in the OSCN dataset is same as those of the CMNIST dataset.

Table S3. The number of images for each shape class in the OSCN dataset.

	Cross	Square	Triangle
**Training**	39,929	40,294	39,777
**Test**	1,337	1,381	1,282

1.3 CMNIST-OSCN

This dataset contains 120,000 image pairs. Each pair contained a 28x28-pixel image from CMNIST and a 32x32-pixel image from OSCN. To create the CMNIST-OSCN, we randomly assigned a corresponding OSCN image to each CMNIST image. For example: if the CMNIST image depicts a white 3, "white 3 cross," "white 3 triangle," or "white 3 squares" are the corresponding OSCN images since they share "white 3." As a result of this process, training (120,000 pairs) and test (4,000 pairs) datasets were constructed. In the analyses of generative ability (reconstruction and cross-generation task) and latent embeddings, 128 images randomly sampled from test dataset were utilized.

2 Model Implementation

2.1 Multi- and single-modal Model

The original implementation of the MMVAE (https://github.com/iffsid/mmvae) was used for the overall model structure. Based on this, we tailored the encoders and decoders for each modality. For the CMNIST, the encoder comprises a single layer with an input/output size of 2,352 (=3x28x28)/400, three layers with an input/output size of 400/400, and one layer with an input/output size of 400/20 (Table S4). Similarly, for the OSCN, the encoder comprises a single layer with an input/output size of 3,072 (=3x32x32)/400, three layers with an input/output size of 400/400, and one layer with an input/output size of 400/20 (Table S5). The decoders have the reversed structures of the corresponding encoder. We determined these model architecture and parameter settings through a parameter search conducted in the preliminary experiments. Objective function was calculated using DReG (a doubly reparameterised gradient) estimator instead of evidence lower bound (Shi et al., 2019).

Table S4. Encoder architecture of the MMVAE model for the CMNIST images.

FC and ReLU represent a fully connected layer and rectified linear unit, respectively.

	Input shape	Output shape

**FC and ReLU**	2,352	400
**FC and ReLU**	400	400
**FC and ReLU**	400	400
**FC and ReLU**	400	400
**FC**	400	20

Table S5. Encoder architecture of the MMVAE model for the OSCN images.

FC and ReLU represent a fully connected layer and rectified linear unit, respectively.

	**Input shape**	**Output shape**
**FC and ReLU**	3,072	400
**FC and ReLU**	400	400
**FC and ReLU**	400	400
**FC and ReLU**	400	400
**FC**	400	20

2.2 Classifier model

To quantitatively analyze whether the networks generate and reconstruct images with accurate number labels, we constructed a classifier model. Two classifier models are implemented using artificial neural networks which can predict the numbers represented by given images of the OSCN and CMNIST datasets. The classifier shares the same architecture as the encoder of single-modal model, with the addition of a fully connected layer predicting number labels at the end. A single classifier model for each modality (OSCN and CMNIST) was trained independent of the MMVAE and VAE model. The dataset for training and test of classifiers was the same as MMVAE. The accuracies at the end of training are 1.000 and 0.977 in the OSCN and CMNIST datasets, respectively.

3 Evaluation Method

3.1 Visualization through dimensionality reduction

To visualize the latent space (originally 20 dimensions) in 2-dimensional space, we used a dimensionality reduction algorithm called t-distributed Stochastic Neighbor Embedding (t-SNE) (van der Maaten & Hinton, 2008). This algorithm can capture similarities and dissimilarities in a high-dimensional space and produce a mapping to low-dimensional spaces in such a way that the relationships between points are preserved. The perplexity parameter was set to 20.

3.2 Silhouette Coefficient

Silhouette coefficient measures the quality of clustering, and it ranges from -1 to 1 (the higher the better). For point p, the silhouette value is defined as:

$$s(p) = \frac{b(p) - a(p)}{max\{a(p), b(p)\}}$$

where $a(p)$ is the average distance between p and all other points in the cluster to which p belongs, and b(p) is the smallest average distance between p and points in other clusters. The value is high when the points in the same cluster are located close together and the points in different clusters are located remotely. We calculated the silhouette coefficient for each data point and the average across all points were used for clustering quality of each model.

3.3 Correlation Coefficients

Firstly, we computed the averages of the latent embeddings for each true number class when images were inputted into the neural network models. The inputs consisted of 128 randomly selected images from the test datasets. Subsequently, we determined the distances between number classes (e.g., the distance between 1 and 3) and the distances between the averaged latent embeddings of each number class (e.g., the distance between averaged latent embeddings when images representing 1 and 3 were used). The distance was defined using L2 norm. These distances were calculated for all possible combinations. Finally, the correlations between both sets of distances were employed as indicators of the quality of the learned number sense.

3.4 Arithmetic Task

In the calculation of success rate in arithmetic task, we used all expression patterns of x + y - z, which satisfy the following conditions.

- x, y, z are integers.
- 1 <= x, y, z <= 9
- 1 <= x + y - z <= 9
- x is not z

As a result of this procedure, 408 expressions were created. The numbers of true answers were as follows, in order from 1 to 9: 36, 43, 48, 51, 52, 51, 48, 43, and 36. Because one image was generated for one expression, the 408 images were analyses for each model.

4 Supplementary Result

4.1 Nonlinearity in latent representations

In the quantitative analysis of numerosity, we employed Pearson's correlation coefficients, assuming linear relationships between variables. However, numerous studies have suggested that numerosity is encoded in neurons using logarithmic transformations rather than linear ones. In this supplementary analysis, we explored the possibility that the latent representations in the multimodal model are nonlinearly associated with numerosity.

In calculating these correlation coefficients, the latent distance (e.g., the distance between the average of points in the latent space belonging to "2" and that of "5") were measured using transformed latent spaces instead of original space. The applied nonlinear transformations included the natural logarithm, exponential, and power of two. Because the latent embeddings take values across the entire real number range, the minimum value of the averaged latent embeddings was added when applying a logarithmic transformation.

We conducted a repeated-measure ANOVA with four levels (Figure S1). The results indicated significant differences between transformations ($F(3, 42) = 125.67$, $p < 0.0001$ in for the OSCN images, and $F(3, 51) = 8.15$, $p = 0.0002$ for the CMNIST images). Post-hoc analysis demonstrated that the correlation coefficients derived from the original latent spaces were either equivalent to or greater than those obtained from transformed spaces in the OSCN dataset ($t(14) = 42.15$; $p < 0.0001$ at original > log, $t(14) = 16.80$; $p < 0.0001$ at original > exp, $t(14) = 11.93$; $p < 0.0001$ at original > pow). Similar findings were observed in the CMNIST dataset ($t(17) = 2.61$; $p = 0.0185$ at original > log, $t(17) = 2.83$; $p = 0.0115$ at original > exp, $t(17) = 3.99$; $p = 0.0009$ at original > pow).

The result suggests that latent embeddings of multimodal models encode numerosity using a linear scale rather than a nonlinear scale.

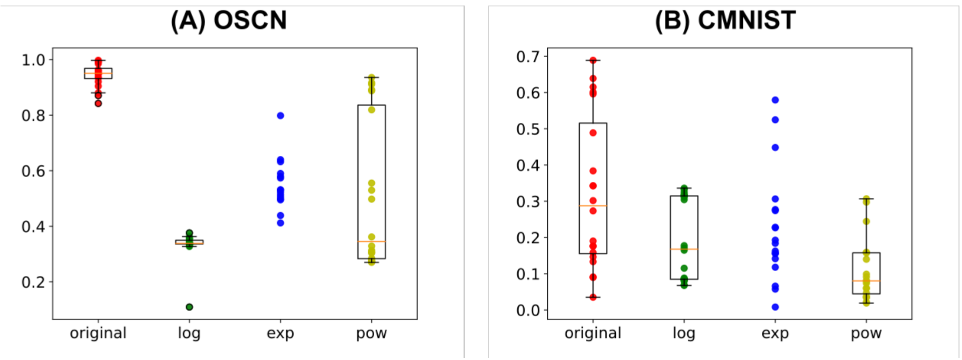

Figure S1. Nonlinearity in latent representations

Nonlinear transformation was applied to latent representations in analyzing correlation coefficiences between the latent distance and the class distance. The terms 'log', 'exp,' and 'pow' in the figure represent the natural logarithm, exponential, and the power of two, respectively. The OSCN (A) and CMNIST (B) datasets.

5 Supplementary Discussion

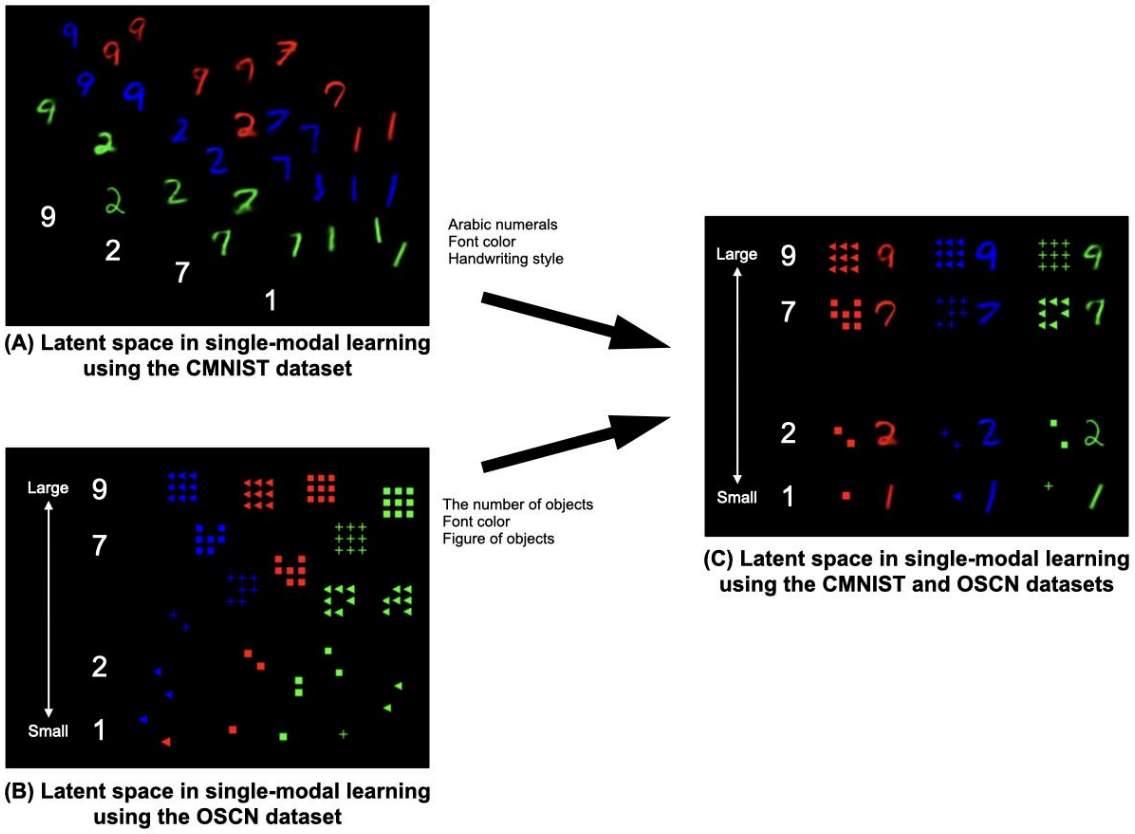

Figure S2. Visualized and conceptual explanation for shared and private latent spaces.

(A) When a neural network model learns numbers using only the CMNIST dataset, its latent space may be organized based on the shapes and colors of hand-written Arabic numerals.

(B) When a neural network model learns numbers using only the OSCN dataset, its latent space may be organized based on the numbers, shapes and colors of objects.

(C) When a neural network model learns numbers using the CMNIST and OSCN dataset, its latent space may be organized based on the shapes and colors of hand-written Arabic numerals in the CMNIST dataset and the numbers, shapes and colors of objects in the OSCN dataset. Numbers and colors carry more information than modality-specific properties since these attributes are present in

both datasets. Consequently, the latent space may primarily be organized using these modality-general properties.

6 References

Shi, Y., Siddharth, N., Paige, B., and Torr, P. H. S. (2019). Variational mixture-of-experts autoencoders for multi-modal deep generative models, in Proceedings of the 33rd International Conference on Neural Information Processing Systems (Curran Associates Inc.), 32.

van der Maaten, L., and Hinton, G. E. (2008). Visualizing data using t-SNE. *J. Mach. Learn. Res*. 9, 2579–2605.
